# The crystal structure of a new ferrocenyl P,N ligand: 1-[(2,2-di­methyl­hydrazin-1-yl­idene)meth­yl]-1′-(di­phenyl­phospho­rothio­yl)ferrocene

**DOI:** 10.1107/S2056989018000440

**Published:** 2018-01-12

**Authors:** Toma Nardjes Mouas, Jean-Claude Daran, Hocine Merazig, Eric Manoury

**Affiliations:** a CNRS, LCC (Laboratoire de Chimie de Coordination), 205 route de Narbonne, BP 44099, F-31077 Toulouse Cedex 4, France; b Unité de Recherche de Chimie de l’Environnement et Molécule Structurales, CHEMS, Université Constantine 1, Algeria; c Département de Biochimie et Bio Mol Cell, Laboratoire de Génétique, Biochimie et Biotechnologie Végétale, Université Constantine 1, Algeria

**Keywords:** crystal structure, ferrocenyl P,*N* ligands, thio­phosphine, hydrazine, catalysis, hydrogen bonding, C—H⋯π inter­actions

## Abstract

The asymmetric unit of the title compound, [Fe(C_8_H_11_N_2_)(C_17_H_14_PS)], contains two independent mol­ecules (*A* and *B*) with very similar conformations. Each mol­ecule is built up from a ferrocene unit substituted in the 1 and 1′ positions by a protected sulfur di­phenyl­phosphine and by a di­methyl­hydrazine, –C(H)=N—N(CH_3_)_2_, fragment. The two independent mol­ecules are linked by a C—H⋯N hydrogen bond. In the crystal, the *A*–*B* dimer is linked by a pair of C—H⋯S hydrogen bonds, forming a centrosymmetric four-mol­ecule arrangement. These units are linked by C—H⋯π inter­actions, forming a supra­molecular three-dimensional structure.

## Abstract 2

The asymmetric unit of the title compound contains two independent mol­ecules with very similar conformations. Each mol­ecule is built up from a ferrocene unit substituted in the 1 and 1′ positions by a protected sulfur di­phenyl­phosphine and by a di­methyl­hydrazine fragment.

## Chemical context   

**Figure 1:**
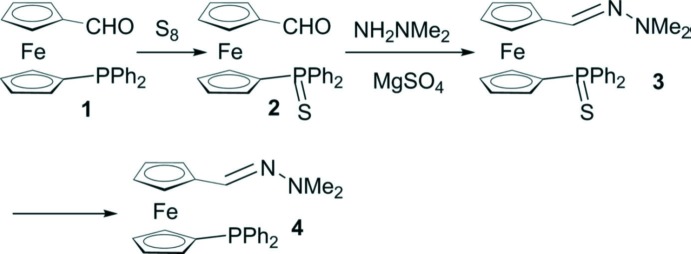
The synthesis of the title compound, **3**.

P,N ligands have proved to be of great inter­est in various fields of catalysis (Börner, 2005 ▸; Carroll & Guiry, 2014 ▸); we were thus inter­ested in obtaining new ferrocenyl P,N ligands (Dwadnia *et al.*, 2018 ▸) bearing both phosphine and hydrazine moieties. Starting from compound **1** (Iftime *et al.*, 1996 ▸), we aimed to obtain target ligand **4** (Fig. 1 ▸). To avoid phosphine oxidation during reactions, work-ups and purifications, the phosphine group was protected as a thio­phosphine by reaction with S_8_ (Routaboul *et al.*, 2005 ▸). The aldehyde-thio­phosphine **2** provided product **3**, the title compound, in one step. A study of the coordination chemistry of the free phosphine **4** and its use in catalytic reactions is now in progress in our laboratory.

## Structural commentary   

**Figure 2:**
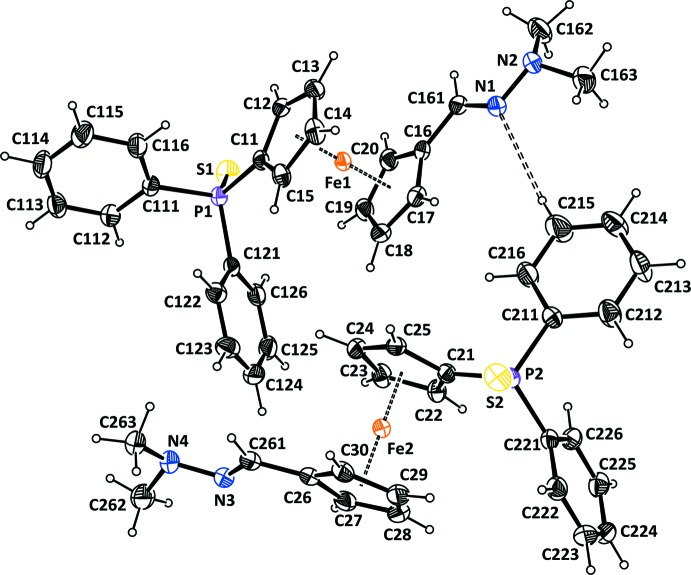
A view of the mol­ecular structures of the two independent mol­ecules (*A* and *B*) of compound **3**, with the atom labelling. Displacement ellipsoids are drawn at the 50% probability level. The C—H⋯N hydrogen bond is shown as a blue dashed line (see Table 2 ▸).

**Figure 3:**
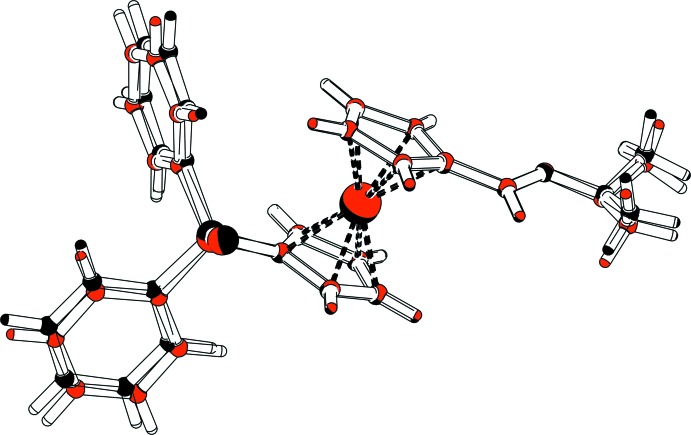
Mol­ecular fitting of the two independent mol­ecules (mol­ecule *A* black and mol­ecule *B* red).

A view of the mol­ecular structures of the two independent mol­ecules (*A* and *B*) of the title compound, **3**, are shown in Fig. 2 ▸. Selected bond lengths and bond angles are given in Table 1 ▸. The two mol­ecules have very similar conformations, as shown in the MolFitView, Fig. 3 ▸ (Spek, 2009 ▸). Each mol­ecule is built up from a ferrocene unit substituted in positions 1,1′ by a protected sulfur di­phenyl­phosphine and by a di­methyl­hydrazine –C(H)=N—N(CH_3_)_2_ fragment. The two independent mol­ecules are linked by a C—H⋯N hydrogen bond (Table 2 ▸ and Fig. 2 ▸).
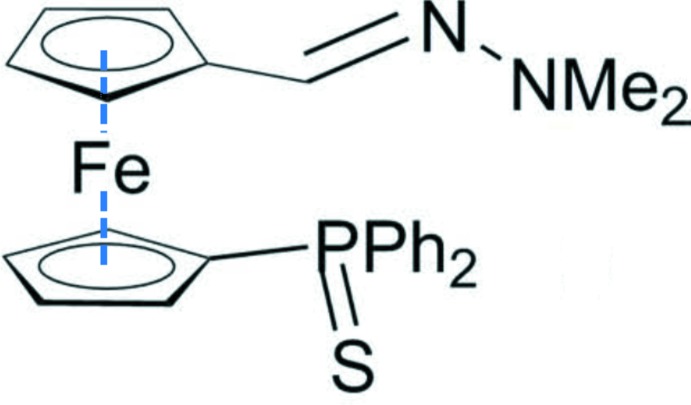


In both mol­ecules, the two Cp rings are between eclipsed and staggered conformations, with a twist angle τ of 5.2 (2)° for mol­ecule *A* and 9.4 (1)° for mol­ecule *B*. However, the Cp rings are roughly parallel to each other with a dihedral angle of 1.46 (12)° for mol­ecule *A* and 1.85 (12)° for mol­ecule *B*. The protected di­phenyl­phosphine and the di­methyl­hydrazine units are approximately *trans* with respect to the ferrocenyl moiety: the torsion angle P1—C11—C16—C161 and P2—C21—C26—C261 are *ca* 140.4° and *ca* 141.0°, respectively.

The sulfur atom is displaced *endo* towards the Fe^II^ ion, by −0.7330 (6) Å (mol­ecule *A*) and 0.6986 (6) Å (mol­ecule *B*) from the Cp ring plane, whereas the phospho­rus atom lies in this plane, displaced by 0.0114 (5) and −0.0603 (4) Å for mol­ecules *A* and *B*, respectively. This arrangement, with the protected sulfur atom *endo* towards the Fe^II^ ion and the P atom roughly coplanar with the Cp ring, is quite common in related compounds (see *Section 4, Database survey*). The geometry within the hydrazine moiety (see Table 1 ▸) is in agreement with already reported structures (Cambridge Structural Database; Groom *et al.*, 2016 ▸). The Cp ring and the substituents on the terminal nitro­gen are in a *trans* position.

## Supra­molecular features   

**Figure 4:**
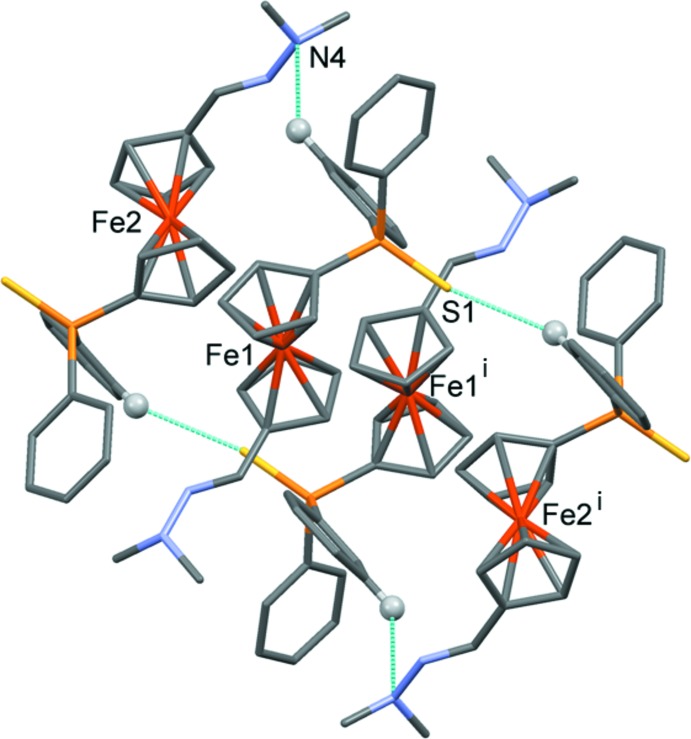
A view of the four-mol­ecule hydrogen-bonded unit. Hydrogen bonds are shown as dashed lines (see Table 2 ▸; only H atoms H123 and H226 have been included). [Symmetry code: (i) −*x*, −*y* + 1, −*z* + 1.]

**Figure 5:**
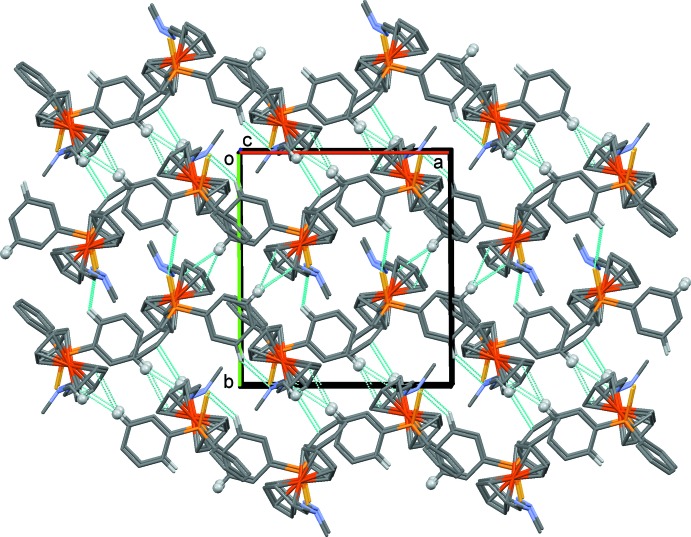
A view along the *c* axis of the crystal packing of compound **3**. The C—H⋯N, C—H⋯S and C—H⋯π inter­actions (see Table 2 ▸) are represented as dashed lines. The H atoms involved in the C—H⋯π inter­actions are shown as grey balls, and only the H atoms involved in the various inter­molecular inter­actions have been included.

In the crystal, the *A*–*B* units are linked through a pair of C—H⋯S hydrogen bonds (Table 2 ▸), forming a four-mol­ecule centrosymmetric unit (Fig. 4 ▸ and Table 2 ▸). These units are linked by C—H⋯π inter­actions, involving the phenyl ring, C111–C116, and the Cp rings, C16–C20 and C26–C30, linking the four-mol­ecule units to form a supra­molecular three-dimensional structure (Fig. 5 ▸ and Table 2 ▸).

## Database survey   

A search of the Cambridge Structural Database (CSD, version 5.38, last update May 2017; Groom *et al.*, 2016 ▸) for ferrocenyl bearing a hydrazine substituent revealed 19 hits, whereas a search using a sulfur-protected di­phenyl­phosphine group resulted in 76 hits. Two of these compounds are of particular inter­est, namely 1,1′-bis­[(2,2-di­methyl­hydrazinyl­idene)meth­yl]ferrocene (CUJDAO; Toma *et al.*, 2015 ▸), which crystallizes with two independent mol­ecules in the asymmetric unit, and 1,1′-bis­(di­phenyl­thio­phosphor­yl)ferrocene, for which two polymorphs have been reported, *viz*. monoclinic *C*2/*c* (ZEQSOD; Fang *et al.*, 1995 ▸) and monoclinic *P*2_1_/*c* (ZEQSOD02; Tan *et al.*, 2015 ▸). In CUJDAO, the substituents are *cis* to one another and in the –C(H)=N—N(CH_3_)_2_ fragments the C=N bond lengths, which vary from 1.270 (5) to 1.287 (4) Å, and the N—N bond lengths, which vary from 1.367 (4) to 1.382 (5) Å, are similar to those in the title compound (see Table 1 ▸).

In ZEQSOD and ZEQSOD02, the P atom is roughly in the Cp ring plane, with deviations from the mean plane ranging from 0.009 (1) to 0.035 (1) Å, whereas the S atom is *endo* towards the Fe^II^ ion with distances ranging from 0.583 (1) to 0.952 (1) Å. The corresponding distances for compound **3** fall within these ranges (see *Section 2, Structural commentary*).

## Synthesis and crystallization   

The synthesis of the title compound, **3**, is illustrated in Fig. 1 ▸. In a Schlenk tube, under argon, were added 66 mg (0.153 mmol) of (1′-di­phenyl­thio­phosphino)ferrocene­carboxaldehyde (**2**), 200 mg (1.66 mmol) of anhydrous magnesium sulfate MgSO_4_ and 5 ml of anhydrous di­chloro­methane. To the red suspension, 100 ml of *N*,*N*-di­methyl­hydrazine (79 mg, 1.31 mmol) was added using a syringe. The reaction mixture was then stirred at room temperature overnight. The crude material obtained was purified by flash chromatography on silica gel to yield 41 mg of compound **3** as a brown solid (yield = 57%). Orange needle-like crystals of **3** were obtained by slow evaporation of a solution in pentane.

Spectroscopic data: ^1^H NMR (400MHz, CDCl_3_): δ (p.p.m.): 7.77–7.71 (*m*, 2H, PPh_2_), 7.49–7.41 (*m*, 8H, PPh_2_), 6.93 (*s*, 1H, CH), 4.57 (*m*, 1H, subst. Cp), 4.49 (*m*, 1H, subst. Cp), 4.36 (*s*, 5H, Cp), 4.20 (*m*, 1H, subst. Cp), 2.79 (*s*, 6H, CH_3_). ^31^P NMR (400MHz, CDCl_3_): δ (p.p.m.): 41.6.

## Refinement   

Crystal data, data collection and structure refinement details are summarized in Table 3 ▸. The C-bound H atoms were included in calculated positions and refined as riding: C—H = 0.95–0.98 Å with *U*_iso_(H) = 1.5*U*_eq_(C-meth­yl) and 1.2*U*_eq_(C) for other H atoms.

## Supplementary Material

Crystal structure: contains datablock(s) I, global. DOI: 10.1107/S2056989018000440/su5416sup1.cif

Structure factors: contains datablock(s) I. DOI: 10.1107/S2056989018000440/su5416Isup2.hkl

CCDC reference: 1815294

Additional supporting information:  crystallographic information; 3D view; checkCIF report

## Figures and Tables

**Table 1:** Selected geometric parameters (Å, °)

S1—P1	1.9525 (7)	N1—C161	1.282 (3)
S2—P2	1.9556 (7)	N3—N4	1.373 (2)
N1—N2	1.378 (2)	N3—C261	1.286 (3)
			
S1—P1—C11	112.75 (7)	N2—N1—C161	119.83 (17)
S2—P2—C21	112.97 (7)	N4—N3—C261	119.70 (17)

**Table 2:** Hydrogen-bond geometry (Å, °) *Cg*1, *Cg*2 and *Cg*3 are the centroids of rings C111–C116, C16–C20 and C26–C30, respectively.

*D*—H⋯*A*	*D*—H	H⋯*A*	*D*⋯*A*	*D*—H⋯*A*
C123—H123⋯N4	0.95	2.62	3.373 (3)	136
C226—H226⋯S1^i^	0.95	2.77	3.416 (2)	126
C262—H26*A*⋯*Cg*1^ii^	0.98	2.91	3.878	172
C125—H125⋯*Cg*2^i^	0.95	2.80	3.533	135
C223—H223⋯*Cg*3^iii^	0.95	2.70	3.478	139

Symmetry codes: (i) 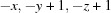; (ii) 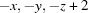; (iii) 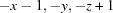.

**Table 3:** Experimental details

Crystal data	
Chemical formula	[Fe(C_8_H_11_N_2_)(C_17_H_14_PS)]
*M*_r_	472.35
Crystal system, space group	Triclinic, *P* 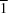
Temperature (K)	180
*a*, *b*, *c* (Å)	12.0684 (5), 13.9953 (4), 14.0634 (5)
α, β, γ (°)	73.682 (3), 86.600 (3), 88.884 (3)
*V* (Å^3^)	2275.61 (14)
*Z*	4
Radiation type	Mo *K*α
μ (mm^−1^)	0.84
Crystal size (mm)	0.36 × 0.12 × 0.04

Data collection	
Diffractometer	Agilent Xcalibur Eos Gemini ultra
Absorption correction	Multi-scan (*CrysAlis PRO*; Agilent, 2012 ▸)
*T*_min_, *T*_max_	0.882, 1.000
No. of measured, independent and observed [*I* > 2σ(*I*)] reflections	49518, 10325, 8270
*R*_int_	0.045
(sin θ/λ)_max_ (Å^−1^)	0.649

Refinement	
*R*[*F* ^2^ > 2σ(*F* ^2^)], *wR*(*F* ^2^), *S*	0.034, 0.080, 1.03
No. of reflections	10325
No. of parameters	545
H-atom treatment	H-atom parameters constrained
Δρ_max_, Δρ_min_ (e Å^−3^)	0.49, −0.24

Computer programs: *CrysAlis PRO* (Agilent, 2012 ▸), *SIR97* (Altomare *et al.*, 1999 ▸), *SHELXL97* (Sheldrick, 2008 ▸), *ORTEP-3 for Windows* (Farrugia, 2012 ▸), *PLATON* (Spek, 2009 ▸), *Mercury* (Macrae *et al.*, 2008 ▸) and *publCIF* (Westrip, 2010 ▸).

## supplementary crystallographic information

### Crystal data

**Table:**

[Fe(C_8_H_11_N_2_)(C_17_H_14_PS)]	*Z* = 4
*M**_r_* = 472.35	*F*(000) = 984
Triclinic, *P*1	*D*_x_ = 1.379 Mg m^−^^3^
Hall symbol: -P 1	Mo *K*α radiation, λ = 0.71073 Å
*a* = 12.0684 (5) Å	Cell parameters from 12406 reflections
*b* = 13.9953 (4) Å	θ = 2.9–29.1°
*c* = 14.0634 (5) Å	µ = 0.84 mm^−^^1^
α = 73.682 (3)°	*T* = 180 K
β = 86.600 (3)°	Needle, orange
γ = 88.884 (3)°	0.36 × 0.12 × 0.04 mm
*V* = 2275.61 (14) Å^3^	

### Data collection

**Table:**

Agilent Xcalibur Eos Gemini ultra diffractometer	10325 independent reflections
Radiation source: Enhance (Mo) X-ray Source	8270 reflections with *I* > 2σ(*I*)
Graphite monochromator	*R*_int_ = 0.045
Detector resolution: 16.1978 pixels mm^-1^	θ_max_ = 27.5°, θ_min_ = 2.9°
ω scans	*h* = −15→15
Absorption correction: multi-scan (CrysAlis PRO; Agilent, 2012)	*k* = −18→17
*T*_min_ = 0.882, *T*_max_ = 1.000	*l* = −18→18
49518 measured reflections	

### Refinement

**Table:**

Refinement on *F*^2^	Primary atom site location: structure-invariant direct methods
Least-squares matrix: full	Secondary atom site location: difference Fourier map
*R*[*F*^2^ > 2σ(*F*^2^)] = 0.034	Hydrogen site location: inferred from neighbouring sites
*wR*(*F*^2^) = 0.080	H-atom parameters constrained
*S* = 1.03	*w* = 1/[σ^2^(*F*_o_^2^) + (0.0318*P*)^2^ + 0.9815*P*] where *P* = (*F*_o_^2^ + 2*F*_c_^2^)/3
10325 reflections	(Δ/σ)_max_ = 0.001
545 parameters	Δρ_max_ = 0.49 e Å^−^^3^
0 restraints	Δρ_min_ = −0.23 e Å^−^^3^

### Special details

**Table:**

Geometry. Bond distances, angles etc. have been calculated using the rounded fractional coordinates. All su's are estimated from the variances of the (full) variance-covariance matrix. The cell esds are taken into account in the estimation of distances, angles and torsion angles
Refinement. Refinement of F^2^ against ALL reflections. The weighted R-factor wR and goodness of fit S are based on F^2^, conventional R-factors R are based on F, with F set to zero for negative F^2^. The threshold expression of F^2^ > 2sigma(F^2^) is used only for calculating R-factors(gt) etc. and is not relevant to the choice of reflections for refinement. R-factors based on F^2^ are statistically about twice as large as those based on F, and R- factors based on ALL data will be even larger.

### Fractional atomic coordinates and isotropic or equivalent isotropic displacement parameters (Å^2^)

**Table:**

	*x*	*y*	*z*	*U*_iso_*/*U*_eq_	
Fe1	0.29555 (2)	0.38485 (2)	0.47919 (2)	0.0212 (1)	
Fe2	−0.20890 (2)	0.11334 (2)	0.53484 (2)	0.0207 (1)	
S1	0.32956 (5)	0.48319 (4)	0.72808 (4)	0.0337 (2)	
P1	0.29834 (4)	0.34682 (3)	0.72809 (3)	0.0197 (1)	
S2	−0.14262 (5)	0.01458 (4)	0.28344 (4)	0.0312 (2)	
P2	−0.17691 (4)	0.15172 (3)	0.28346 (3)	0.0199 (1)	
N1	0.31107 (13)	0.47934 (12)	0.17859 (12)	0.0263 (5)	
N2	0.37378 (14)	0.50966 (13)	0.09033 (12)	0.0300 (5)	
C11	0.34891 (15)	0.31978 (13)	0.61637 (13)	0.0207 (5)	
C12	0.43773 (15)	0.37360 (14)	0.55323 (14)	0.0250 (6)	
C13	0.45558 (17)	0.33332 (15)	0.47176 (15)	0.0306 (6)	
C14	0.37924 (18)	0.25475 (15)	0.48349 (14)	0.0302 (6)	
C15	0.31316 (17)	0.24542 (13)	0.57263 (13)	0.0247 (6)	
C16	0.26428 (16)	0.48251 (14)	0.34328 (13)	0.0243 (6)	
C17	0.18363 (17)	0.40472 (16)	0.37151 (15)	0.0311 (7)	
C18	0.12905 (17)	0.40871 (17)	0.46303 (15)	0.0341 (7)	
C19	0.17646 (17)	0.48726 (16)	0.49212 (15)	0.0316 (6)	
C20	0.26004 (17)	0.53223 (14)	0.41921 (14)	0.0273 (6)	
C111	0.36505 (15)	0.25668 (13)	0.82715 (13)	0.0221 (5)	
C112	0.32953 (17)	0.24922 (15)	0.92498 (14)	0.0286 (6)	
C113	0.38548 (19)	0.18785 (16)	1.00229 (15)	0.0351 (7)	
C114	0.47719 (19)	0.13462 (17)	0.98267 (16)	0.0388 (7)	
C115	0.5117 (2)	0.14036 (18)	0.88618 (17)	0.0414 (8)	
C116	0.45546 (18)	0.20130 (16)	0.80824 (15)	0.0331 (7)	
C121	0.15268 (15)	0.31491 (14)	0.74696 (13)	0.0218 (6)	
C122	0.12050 (17)	0.21554 (15)	0.76907 (14)	0.0261 (6)	
C123	0.00931 (18)	0.19092 (17)	0.78017 (15)	0.0340 (7)	
C124	−0.07030 (18)	0.26496 (19)	0.76967 (15)	0.0396 (8)	
C125	−0.03898 (19)	0.36327 (19)	0.74910 (15)	0.0393 (7)	
C126	0.07256 (17)	0.38880 (16)	0.73858 (14)	0.0297 (6)	
C161	0.33223 (16)	0.51348 (14)	0.25108 (14)	0.0252 (6)	
C162	0.4271 (2)	0.60605 (18)	0.06628 (17)	0.0452 (8)	
C163	0.3132 (2)	0.49281 (19)	0.00988 (16)	0.0417 (8)	
N3	−0.21735 (13)	0.02865 (12)	0.83405 (12)	0.0283 (5)	
N4	−0.15754 (14)	0.00672 (14)	0.91759 (12)	0.0331 (6)	
C21	−0.14436 (15)	0.17776 (13)	0.39595 (13)	0.0205 (5)	
C22	−0.18894 (16)	0.25245 (13)	0.43929 (13)	0.0238 (6)	
C23	−0.13150 (17)	0.24494 (14)	0.52684 (14)	0.0281 (6)	
C24	−0.05230 (16)	0.16746 (15)	0.53756 (14)	0.0283 (6)	
C25	−0.05905 (15)	0.12595 (14)	0.45703 (14)	0.0237 (6)	
C26	−0.25004 (16)	0.01668 (14)	0.67335 (14)	0.0251 (6)	
C27	−0.33138 (16)	0.09358 (16)	0.64521 (15)	0.0302 (6)	
C28	−0.37565 (17)	0.08723 (17)	0.55543 (16)	0.0349 (7)	
C29	−0.32141 (17)	0.00876 (16)	0.52678 (15)	0.0321 (7)	
C30	−0.24360 (17)	−0.03440 (14)	0.59817 (14)	0.0275 (6)	
C211	−0.09704 (16)	0.24208 (14)	0.18730 (14)	0.0235 (5)	
C212	−0.1180 (2)	0.25478 (16)	0.08900 (15)	0.0368 (7)	
C213	−0.0538 (2)	0.31903 (18)	0.01409 (16)	0.0446 (8)	
C214	0.0313 (2)	0.37062 (17)	0.03698 (17)	0.0428 (8)	
C215	0.0525 (2)	0.3595 (2)	0.13394 (19)	0.0579 (10)	
C216	−0.0117 (2)	0.29510 (19)	0.20944 (16)	0.0446 (8)	
C221	−0.32018 (15)	0.18623 (14)	0.26159 (13)	0.0220 (6)	
C222	−0.39991 (17)	0.11370 (15)	0.26912 (14)	0.0299 (6)	
C223	−0.50987 (18)	0.14120 (17)	0.25456 (16)	0.0371 (7)	
C224	−0.54057 (17)	0.23994 (18)	0.23257 (15)	0.0352 (7)	
C225	−0.46146 (17)	0.31273 (16)	0.22343 (15)	0.0307 (6)	
C226	−0.35161 (16)	0.28597 (14)	0.23745 (14)	0.0255 (6)	
C261	−0.18802 (16)	−0.00903 (15)	0.76283 (14)	0.0268 (6)	
C262	−0.21902 (19)	0.02841 (19)	1.00094 (16)	0.0400 (8)	
C263	−0.08752 (18)	−0.08097 (18)	0.93953 (16)	0.0385 (7)	
H12	0.47774	0.42703	0.56406	0.0300*	
H13	0.50955	0.35526	0.41838	0.0370*	
H14	0.37323	0.21502	0.43927	0.0360*	
H15	0.25559	0.19836	0.59861	0.0300*	
H16A	0.37178	0.65686	0.07142	0.0680*	
H16B	0.46040	0.62226	−0.00158	0.0680*	
H16C	0.48500	0.60423	0.11272	0.0680*	
H16D	0.28397	0.42481	0.02980	0.0630*	
H16E	0.36336	0.50193	−0.04938	0.0630*	
H16F	0.25161	0.54038	−0.00487	0.0630*	
H17	0.16898	0.35852	0.33562	0.0370*	
H18	0.07097	0.36610	0.49839	0.0410*	
H19	0.15576	0.50642	0.55040	0.0380*	
H20	0.30560	0.58636	0.42060	0.0330*	
H112	0.26683	0.28630	0.93867	0.0340*	
H113	0.36068	0.18236	1.06899	0.0420*	
H114	0.51660	0.09392	1.03586	0.0470*	
H115	0.57404	0.10265	0.87288	0.0500*	
H116	0.47921	0.20491	0.74170	0.0400*	
H122	0.17523	0.16466	0.77651	0.0310*	
H123	−0.01243	0.12321	0.79500	0.0410*	
H124	−0.14680	0.24810	0.77665	0.0480*	
H125	−0.09408	0.41381	0.74209	0.0470*	
H126	0.09388	0.45643	0.72573	0.0360*	
H161	0.39181	0.55865	0.24462	0.0300*	
H22	−0.24626	0.29855	0.41429	0.0290*	
H23	−0.14433	0.28527	0.57063	0.0340*	
H24	−0.00301	0.14676	0.58977	0.0340*	
H25	−0.01471	0.07301	0.44559	0.0280*	
H26A	−0.27379	−0.02412	1.02954	0.0600*	
H26B	−0.25719	0.09255	0.97818	0.0600*	
H26C	−0.16762	0.03145	1.05139	0.0600*	
H26D	−0.02544	−0.07165	0.88965	0.0580*	
H26E	−0.13099	−0.13902	0.93838	0.0580*	
H26F	−0.05870	−0.09163	1.00542	0.0580*	
H27	−0.35229	0.14069	0.68024	0.0360*	
H28	−0.43204	0.12877	0.52085	0.0420*	
H29	−0.33475	−0.01163	0.46950	0.0390*	
H30	−0.19517	−0.08829	0.59645	0.0330*	
H212	−0.17686	0.21920	0.07269	0.0440*	
H213	−0.06871	0.32737	−0.05333	0.0530*	
H214	0.07564	0.41420	−0.01460	0.0510*	
H215	0.11106	0.39571	0.14970	0.0690*	
H216	0.00321	0.28755	0.27674	0.0540*	
H222	−0.37905	0.04551	0.28420	0.0360*	
H223	−0.56439	0.09166	0.25978	0.0450*	
H224	−0.61629	0.25815	0.22364	0.0420*	
H225	−0.48266	0.38087	0.20757	0.0370*	
H226	−0.29718	0.33595	0.23057	0.0310*	
H261	−0.12645	−0.05323	0.76856	0.0320*	

### Atomic displacement parameters (Å^2^)

**Table:**

	*U*^11^	*U*^22^	*U*^33^	*U*^12^	*U*^13^	*U*^23^
Fe1	0.0222 (1)	0.0216 (1)	0.0175 (1)	0.0012 (1)	−0.0012 (1)	−0.0020 (1)
Fe2	0.0207 (1)	0.0215 (1)	0.0181 (1)	−0.0004 (1)	−0.0013 (1)	−0.0027 (1)
S1	0.0460 (3)	0.0199 (2)	0.0377 (3)	−0.0039 (2)	−0.0072 (2)	−0.0109 (2)
P1	0.0236 (2)	0.0167 (2)	0.0189 (2)	0.0000 (2)	−0.0029 (2)	−0.0049 (2)
S2	0.0404 (3)	0.0200 (2)	0.0341 (3)	0.0031 (2)	0.0015 (2)	−0.0101 (2)
P2	0.0235 (2)	0.0172 (2)	0.0188 (2)	−0.0007 (2)	−0.0009 (2)	−0.0046 (2)
N1	0.0249 (9)	0.0292 (9)	0.0222 (8)	0.0013 (7)	−0.0013 (7)	−0.0032 (7)
N2	0.0290 (9)	0.0394 (10)	0.0202 (8)	−0.0016 (8)	0.0003 (7)	−0.0063 (7)
C11	0.0237 (10)	0.0187 (9)	0.0174 (9)	0.0026 (7)	−0.0028 (7)	−0.0014 (7)
C12	0.0197 (10)	0.0273 (10)	0.0241 (10)	0.0016 (8)	−0.0038 (7)	−0.0006 (8)
C13	0.0266 (11)	0.0351 (11)	0.0241 (10)	0.0099 (9)	0.0031 (8)	0.0001 (9)
C14	0.0420 (13)	0.0255 (10)	0.0229 (10)	0.0096 (9)	−0.0016 (9)	−0.0074 (8)
C15	0.0331 (11)	0.0182 (9)	0.0210 (9)	0.0017 (8)	−0.0024 (8)	−0.0027 (7)
C16	0.0225 (10)	0.0276 (10)	0.0196 (9)	0.0036 (8)	−0.0054 (7)	−0.0006 (8)
C17	0.0255 (11)	0.0373 (12)	0.0266 (11)	−0.0040 (9)	−0.0070 (8)	−0.0012 (9)
C18	0.0204 (10)	0.0435 (13)	0.0301 (11)	0.0009 (9)	−0.0025 (8)	0.0033 (9)
C19	0.0304 (11)	0.0367 (12)	0.0228 (10)	0.0134 (9)	−0.0005 (8)	−0.0016 (9)
C20	0.0295 (11)	0.0245 (10)	0.0250 (10)	0.0063 (8)	−0.0039 (8)	−0.0024 (8)
C111	0.0248 (10)	0.0205 (9)	0.0208 (9)	−0.0009 (7)	−0.0055 (7)	−0.0047 (7)
C112	0.0303 (11)	0.0298 (11)	0.0259 (10)	0.0006 (8)	−0.0041 (8)	−0.0079 (8)
C113	0.0435 (13)	0.0400 (12)	0.0205 (10)	−0.0037 (10)	−0.0054 (9)	−0.0056 (9)
C114	0.0420 (13)	0.0389 (13)	0.0311 (12)	0.0041 (10)	−0.0145 (10)	−0.0002 (10)
C115	0.0378 (13)	0.0451 (14)	0.0378 (13)	0.0152 (10)	−0.0077 (10)	−0.0056 (10)
C116	0.0350 (12)	0.0381 (12)	0.0238 (10)	0.0095 (9)	−0.0038 (9)	−0.0051 (9)
C121	0.0237 (10)	0.0264 (10)	0.0150 (9)	0.0011 (8)	−0.0011 (7)	−0.0055 (7)
C122	0.0281 (10)	0.0285 (10)	0.0233 (10)	−0.0015 (8)	0.0008 (8)	−0.0104 (8)
C123	0.0316 (12)	0.0438 (13)	0.0301 (11)	−0.0115 (10)	0.0025 (9)	−0.0163 (10)
C124	0.0260 (11)	0.0677 (17)	0.0249 (11)	−0.0062 (11)	−0.0014 (9)	−0.0123 (11)
C125	0.0310 (12)	0.0574 (15)	0.0242 (11)	0.0174 (11)	−0.0038 (9)	−0.0034 (10)
C126	0.0320 (11)	0.0315 (11)	0.0212 (10)	0.0074 (9)	0.0000 (8)	−0.0010 (8)
C161	0.0213 (10)	0.0270 (10)	0.0240 (10)	0.0013 (8)	−0.0027 (8)	−0.0015 (8)
C162	0.0500 (15)	0.0543 (15)	0.0275 (12)	−0.0224 (12)	0.0059 (10)	−0.0054 (11)
C163	0.0456 (14)	0.0535 (15)	0.0280 (12)	−0.0057 (11)	−0.0015 (10)	−0.0144 (11)
N3	0.0243 (9)	0.0334 (9)	0.0240 (9)	−0.0045 (7)	−0.0017 (7)	−0.0027 (7)
N4	0.0296 (10)	0.0448 (11)	0.0220 (9)	−0.0011 (8)	−0.0040 (7)	−0.0042 (8)
C21	0.0221 (9)	0.0174 (9)	0.0200 (9)	−0.0017 (7)	−0.0014 (7)	−0.0019 (7)
C22	0.0310 (11)	0.0183 (9)	0.0217 (9)	0.0012 (8)	−0.0044 (8)	−0.0043 (7)
C23	0.0384 (12)	0.0245 (10)	0.0225 (10)	−0.0055 (9)	−0.0040 (8)	−0.0078 (8)
C24	0.0252 (10)	0.0321 (11)	0.0242 (10)	−0.0071 (8)	−0.0055 (8)	−0.0009 (8)
C25	0.0189 (9)	0.0242 (10)	0.0242 (10)	0.0000 (7)	−0.0006 (7)	−0.0008 (8)
C26	0.0205 (10)	0.0294 (10)	0.0218 (9)	−0.0043 (8)	0.0022 (7)	−0.0015 (8)
C27	0.0230 (10)	0.0364 (12)	0.0270 (11)	0.0029 (8)	0.0057 (8)	−0.0036 (9)
C28	0.0203 (10)	0.0443 (13)	0.0330 (12)	−0.0018 (9)	−0.0031 (8)	0.0013 (10)
C29	0.0297 (11)	0.0362 (12)	0.0282 (11)	−0.0144 (9)	−0.0022 (8)	−0.0043 (9)
C30	0.0287 (11)	0.0245 (10)	0.0256 (10)	−0.0065 (8)	0.0021 (8)	−0.0011 (8)
C211	0.0270 (10)	0.0210 (9)	0.0213 (9)	0.0000 (8)	0.0009 (8)	−0.0043 (7)
C212	0.0491 (14)	0.0369 (12)	0.0255 (11)	−0.0110 (10)	−0.0011 (10)	−0.0101 (9)
C213	0.0643 (17)	0.0475 (14)	0.0188 (11)	−0.0084 (12)	0.0027 (10)	−0.0048 (10)
C214	0.0503 (15)	0.0398 (13)	0.0313 (12)	−0.0119 (11)	0.0106 (10)	−0.0006 (10)
C215	0.0593 (17)	0.0739 (19)	0.0371 (14)	−0.0416 (15)	0.0031 (12)	−0.0082 (13)
C216	0.0468 (14)	0.0601 (16)	0.0230 (11)	−0.0266 (12)	−0.0019 (10)	−0.0034 (10)
C221	0.0240 (10)	0.0251 (10)	0.0172 (9)	−0.0014 (8)	−0.0025 (7)	−0.0059 (7)
C222	0.0348 (12)	0.0270 (10)	0.0254 (10)	−0.0073 (9)	−0.0048 (8)	−0.0023 (8)
C223	0.0296 (12)	0.0474 (14)	0.0316 (12)	−0.0151 (10)	−0.0028 (9)	−0.0054 (10)
C224	0.0217 (11)	0.0582 (15)	0.0257 (11)	0.0010 (10)	−0.0034 (8)	−0.0116 (10)
C225	0.0321 (11)	0.0356 (12)	0.0265 (10)	0.0074 (9)	−0.0055 (8)	−0.0119 (9)
C226	0.0264 (10)	0.0261 (10)	0.0259 (10)	−0.0007 (8)	−0.0041 (8)	−0.0098 (8)
C261	0.0214 (10)	0.0297 (11)	0.0253 (10)	−0.0019 (8)	0.0016 (8)	−0.0016 (8)
C262	0.0387 (13)	0.0551 (15)	0.0303 (12)	−0.0046 (11)	−0.0043 (10)	−0.0177 (11)
C263	0.0311 (12)	0.0526 (14)	0.0268 (11)	0.0023 (10)	−0.0061 (9)	−0.0021 (10)

### Geometric parameters (Å, º)

**Table:**

Fe1—C11	2.0256 (18)	C20—H20	0.9500
Fe1—C12	2.0402 (19)	C112—H112	0.9500
Fe1—C13	2.056 (2)	C113—H113	0.9500
Fe1—C14	2.053 (2)	C114—H114	0.9500
Fe1—C15	2.0386 (19)	C115—H115	0.9500
Fe1—C16	2.0643 (18)	C116—H116	0.9500
Fe1—C17	2.046 (2)	C122—H122	0.9500
Fe1—C18	2.046 (2)	C123—H123	0.9500
Fe1—C19	2.047 (2)	C124—H124	0.9500
Fe1—C20	2.045 (2)	C125—H125	0.9500
Fe2—C28	2.041 (2)	C126—H126	0.9500
Fe2—C29	2.046 (2)	C161—H161	0.9500
Fe2—C30	2.049 (2)	C162—H16C	0.9800
Fe2—C21	2.0229 (18)	C162—H16A	0.9800
Fe2—C22	2.0406 (19)	C162—H16B	0.9800
Fe2—C23	2.053 (2)	C163—H16D	0.9800
Fe2—C24	2.056 (2)	C163—H16E	0.9800
Fe2—C25	2.0416 (19)	C163—H16F	0.9800
Fe2—C26	2.0740 (19)	C21—C22	1.432 (3)
Fe2—C27	2.043 (2)	C21—C25	1.432 (3)
S1—P1	1.9525 (7)	C22—C23	1.426 (3)
P1—C111	1.8128 (19)	C23—C24	1.413 (3)
P1—C11	1.7876 (18)	C24—C25	1.418 (3)
P1—C121	1.8080 (19)	C26—C27	1.430 (3)
S2—P2	1.9556 (7)	C26—C30	1.431 (3)
P2—C221	1.8068 (19)	C26—C261	1.456 (3)
P2—C211	1.814 (2)	C27—C28	1.426 (3)
P2—C21	1.7869 (18)	C28—C29	1.409 (3)
N1—N2	1.378 (2)	C29—C30	1.416 (3)
N1—C161	1.282 (3)	C211—C212	1.381 (3)
N2—C163	1.458 (3)	C211—C216	1.381 (3)
N2—C162	1.449 (3)	C212—C213	1.385 (3)
C11—C15	1.434 (3)	C213—C214	1.371 (3)
C11—C12	1.431 (3)	C214—C215	1.367 (3)
C12—C13	1.416 (3)	C215—C216	1.390 (3)
C13—C14	1.416 (3)	C221—C222	1.390 (3)
C14—C15	1.421 (3)	C221—C226	1.391 (3)
C16—C161	1.454 (3)	C222—C223	1.385 (3)
C16—C17	1.429 (3)	C223—C224	1.377 (4)
C16—C20	1.427 (3)	C224—C225	1.384 (3)
C17—C18	1.426 (3)	C225—C226	1.381 (3)
C18—C19	1.417 (3)	C22—H22	0.9500
C19—C20	1.416 (3)	C23—H23	0.9500
C111—C112	1.392 (3)	C24—H24	0.9500
C111—C116	1.382 (3)	C25—H25	0.9500
C112—C113	1.384 (3)	C27—H27	0.9500
C113—C114	1.380 (3)	C28—H28	0.9500
C114—C115	1.376 (3)	C29—H29	0.9500
C115—C116	1.389 (3)	C30—H30	0.9500
C121—C126	1.387 (3)	C212—H212	0.9500
C121—C122	1.395 (3)	C213—H213	0.9500
C122—C123	1.381 (3)	C214—H214	0.9500
C123—C124	1.382 (3)	C215—H215	0.9500
C124—C125	1.380 (4)	C216—H216	0.9500
C125—C126	1.388 (3)	C222—H222	0.9500
N3—N4	1.373 (2)	C223—H223	0.9500
N3—C261	1.286 (3)	C224—H224	0.9500
N4—C263	1.447 (3)	C225—H225	0.9500
N4—C262	1.451 (3)	C226—H226	0.9500
C12—H12	0.9500	C261—H261	0.9500
C13—H13	0.9500	C262—H26A	0.9800
C14—H14	0.9500	C262—H26B	0.9800
C15—H15	0.9500	C262—H26C	0.9800
C17—H17	0.9500	C263—H26D	0.9800
C18—H18	0.9500	C263—H26E	0.9800
C19—H19	0.9500	C263—H26F	0.9800
			
C11—Fe1—C12	41.21 (7)	Fe1—C13—H13	127.00
C11—Fe1—C13	68.76 (8)	C12—C13—H13	126.00
C11—Fe1—C14	68.85 (8)	C14—C13—H13	126.00
C11—Fe1—C15	41.32 (7)	Fe1—C14—H14	127.00
C11—Fe1—C16	164.93 (8)	C13—C14—H14	126.00
C11—Fe1—C17	152.95 (8)	C15—C14—H14	126.00
C11—Fe1—C18	118.79 (8)	Fe1—C15—H15	126.00
C11—Fe1—C19	107.75 (8)	C11—C15—H15	126.00
C11—Fe1—C20	127.05 (8)	C14—C15—H15	126.00
C12—Fe1—C13	40.46 (8)	Fe1—C17—H17	126.00
C12—Fe1—C14	68.25 (8)	C16—C17—H17	126.00
C12—Fe1—C15	68.98 (8)	C18—C17—H17	126.00
C12—Fe1—C16	127.08 (8)	C19—C18—H18	126.00
C12—Fe1—C17	164.06 (8)	Fe1—C18—H18	126.00
C12—Fe1—C18	154.23 (8)	C17—C18—H18	126.00
C12—Fe1—C19	120.34 (8)	C18—C19—H19	126.00
C12—Fe1—C20	108.73 (8)	C20—C19—H19	126.00
C13—Fe1—C14	40.32 (9)	Fe1—C19—H19	126.00
C13—Fe1—C15	68.38 (8)	Fe1—C20—H20	126.00
C13—Fe1—C16	108.23 (8)	C16—C20—H20	126.00
C13—Fe1—C17	126.27 (8)	C19—C20—H20	126.00
C13—Fe1—C18	163.59 (9)	C111—C112—H112	120.00
C13—Fe1—C19	154.71 (9)	C113—C112—H112	120.00
C13—Fe1—C20	120.41 (8)	C112—C113—H113	120.00
C14—Fe1—C15	40.64 (8)	C114—C113—H113	120.00
C14—Fe1—C16	119.09 (8)	C113—C114—H114	120.00
C14—Fe1—C17	106.96 (9)	C115—C114—H114	120.00
C14—Fe1—C18	126.07 (9)	C116—C115—H115	120.00
C14—Fe1—C19	163.82 (9)	C114—C115—H115	120.00
C14—Fe1—C20	153.92 (8)	C115—C116—H116	120.00
C15—Fe1—C16	152.71 (8)	C111—C116—H116	120.00
C15—Fe1—C17	118.01 (9)	C121—C122—H122	120.00
C15—Fe1—C18	106.88 (9)	C123—C122—H122	120.00
C15—Fe1—C19	126.52 (8)	C122—C123—H123	120.00
C15—Fe1—C20	164.66 (8)	C124—C123—H123	120.00
C16—Fe1—C17	40.69 (8)	C123—C124—H124	120.00
C16—Fe1—C18	68.33 (8)	C125—C124—H124	120.00
C16—Fe1—C19	68.27 (8)	C124—C125—H125	120.00
C16—Fe1—C20	40.65 (8)	C126—C125—H125	120.00
C17—Fe1—C18	40.77 (8)	C125—C126—H126	120.00
C17—Fe1—C19	68.45 (9)	C121—C126—H126	120.00
C17—Fe1—C20	68.46 (9)	N1—C161—H161	120.00
C18—Fe1—C19	40.52 (9)	C16—C161—H161	120.00
C18—Fe1—C20	68.20 (9)	H16A—C162—H16C	109.00
C19—Fe1—C20	40.49 (8)	N2—C162—H16A	109.00
C21—Fe2—C28	118.70 (8)	N2—C162—H16B	109.00
C21—Fe2—C29	107.94 (8)	H16B—C162—H16C	110.00
C21—Fe2—C30	127.42 (8)	H16A—C162—H16B	109.00
C22—Fe2—C23	40.77 (7)	N2—C162—H16C	109.00
C22—Fe2—C24	68.49 (8)	N2—C163—H16E	109.00
C22—Fe2—C25	68.98 (8)	N2—C163—H16F	109.00
C22—Fe2—C26	152.45 (8)	H16D—C163—H16F	109.00
C22—Fe2—C27	117.80 (8)	H16E—C163—H16F	109.00
C22—Fe2—C28	106.78 (9)	H16D—C163—H16E	109.00
C22—Fe2—C29	126.53 (8)	N2—C163—H16D	109.00
C22—Fe2—C30	164.80 (8)	Fe2—C21—P2	127.79 (10)
C23—Fe2—C24	40.23 (8)	Fe2—C21—C22	70.03 (10)
C23—Fe2—C25	68.14 (8)	Fe2—C21—C25	70.08 (10)
C23—Fe2—C26	118.85 (8)	P2—C21—C22	129.53 (14)
C23—Fe2—C27	106.84 (9)	P2—C21—C25	122.73 (14)
C23—Fe2—C28	126.17 (9)	C22—C21—C25	107.66 (16)
C23—Fe2—C29	163.87 (8)	Fe2—C22—C21	68.70 (10)
C23—Fe2—C30	153.77 (8)	Fe2—C22—C23	70.09 (11)
C24—Fe2—C25	40.47 (8)	C21—C22—C23	107.45 (16)
C24—Fe2—C26	108.15 (8)	Fe2—C23—C22	69.14 (11)
C24—Fe2—C27	126.15 (8)	Fe2—C23—C24	70.00 (12)
C24—Fe2—C28	163.60 (9)	C22—C23—C24	108.58 (17)
C24—Fe2—C29	154.85 (9)	Fe2—C24—C23	69.77 (11)
C24—Fe2—C30	120.52 (8)	Fe2—C24—C25	69.21 (11)
C25—Fe2—C26	127.26 (8)	C23—C24—C25	108.26 (17)
C25—Fe2—C27	164.09 (8)	Fe2—C25—C21	68.68 (10)
C25—Fe2—C28	154.19 (8)	Fe2—C25—C24	70.31 (11)
C25—Fe2—C29	120.58 (8)	C21—C25—C24	108.05 (17)
C25—Fe2—C30	109.10 (8)	Fe2—C26—C27	68.51 (11)
C26—Fe2—C27	40.63 (8)	Fe2—C26—C30	68.76 (11)
C26—Fe2—C28	68.34 (8)	Fe2—C26—C261	129.59 (14)
C26—Fe2—C29	68.26 (8)	C27—C26—C30	106.83 (17)
C26—Fe2—C30	40.61 (8)	C27—C26—C261	127.52 (18)
C27—Fe2—C28	40.86 (8)	C30—C26—C261	125.61 (18)
C27—Fe2—C29	68.37 (9)	Fe2—C27—C26	70.86 (11)
C27—Fe2—C30	68.30 (9)	Fe2—C27—C28	69.52 (12)
C28—Fe2—C29	40.34 (9)	C26—C27—C28	108.10 (19)
C28—Fe2—C30	68.00 (9)	Fe2—C28—C27	69.63 (12)
C29—Fe2—C30	40.44 (8)	Fe2—C28—C29	70.02 (12)
C21—Fe2—C25	41.24 (7)	C27—C28—C29	108.28 (19)
C21—Fe2—C26	165.24 (8)	Fe2—C29—C28	69.64 (13)
C21—Fe2—C27	152.78 (8)	Fe2—C29—C30	69.88 (12)
C21—Fe2—C24	68.84 (7)	C28—C29—C30	108.13 (18)
C21—Fe2—C22	41.27 (7)	Fe2—C30—C26	70.63 (11)
C21—Fe2—C23	68.84 (8)	Fe2—C30—C29	69.68 (12)
S1—P1—C11	112.75 (7)	C26—C30—C29	108.63 (18)
C111—P1—C121	104.98 (8)	P2—C211—C212	119.41 (16)
S1—P1—C111	111.65 (7)	P2—C211—C216	121.64 (15)
S1—P1—C121	113.99 (7)	C212—C211—C216	118.88 (19)
C11—P1—C111	105.33 (9)	C211—C212—C213	120.4 (2)
C11—P1—C121	107.48 (9)	C212—C213—C214	120.2 (2)
S2—P2—C221	113.62 (7)	C213—C214—C215	120.1 (2)
C21—P2—C211	104.31 (9)	C214—C215—C216	120.0 (2)
S2—P2—C211	112.51 (7)	C211—C216—C215	120.5 (2)
S2—P2—C21	112.97 (7)	P2—C221—C222	120.50 (15)
C21—P2—C221	107.76 (9)	P2—C221—C226	120.05 (15)
C211—P2—C221	104.95 (9)	C222—C221—C226	119.45 (18)
N2—N1—C161	119.83 (17)	C221—C222—C223	119.8 (2)
N1—N2—C163	110.15 (16)	C222—C223—C224	120.4 (2)
N1—N2—C162	117.80 (17)	C223—C224—C225	120.2 (2)
C162—N2—C163	114.11 (17)	C224—C225—C226	119.8 (2)
P1—C11—C12	123.06 (14)	C221—C226—C225	120.39 (19)
P1—C11—C15	129.50 (14)	N3—C261—C26	119.40 (18)
Fe1—C11—C15	69.83 (10)	Fe2—C22—H22	126.00
Fe1—C11—P1	125.49 (10)	C21—C22—H22	126.00
Fe1—C11—C12	69.94 (10)	C23—C22—H22	126.00
C12—C11—C15	107.44 (16)	Fe2—C23—H23	127.00
Fe1—C12—C11	68.85 (10)	C22—C23—H23	126.00
Fe1—C12—C13	70.35 (11)	C24—C23—H23	126.00
C11—C12—C13	108.08 (17)	Fe2—C24—H24	127.00
C12—C13—C14	108.33 (17)	C23—C24—H24	126.00
Fe1—C13—C12	69.19 (11)	C25—C24—H24	126.00
Fe1—C13—C14	69.73 (12)	Fe2—C25—H25	127.00
Fe1—C14—C13	69.95 (12)	C21—C25—H25	126.00
Fe1—C14—C15	69.16 (12)	C24—C25—H25	126.00
C13—C14—C15	108.40 (18)	Fe2—C27—H27	125.00
C11—C15—C14	107.75 (17)	C26—C27—H27	126.00
Fe1—C15—C14	70.21 (11)	C28—C27—H27	126.00
Fe1—C15—C11	68.86 (10)	Fe2—C28—H28	126.00
Fe1—C16—C20	68.94 (11)	C27—C28—H28	126.00
Fe1—C16—C17	68.97 (11)	C29—C28—H28	126.00
C17—C16—C161	127.72 (18)	Fe2—C29—H29	126.00
Fe1—C16—C161	131.42 (14)	C28—C29—H29	126.00
C17—C16—C20	107.34 (17)	C30—C29—H29	126.00
C20—C16—C161	124.73 (18)	Fe2—C30—H30	126.00
Fe1—C17—C18	69.61 (12)	C26—C30—H30	126.00
C16—C17—C18	107.92 (19)	C29—C30—H30	126.00
Fe1—C17—C16	70.34 (11)	C211—C212—H212	120.00
C17—C18—C19	108.15 (19)	C213—C212—H212	120.00
Fe1—C18—C17	69.62 (12)	C212—C213—H213	120.00
Fe1—C18—C19	69.76 (12)	C214—C213—H213	120.00
C18—C19—C20	108.10 (18)	C213—C214—H214	120.00
Fe1—C19—C20	69.68 (12)	C215—C214—H214	120.00
Fe1—C19—C18	69.72 (13)	C214—C215—H215	120.00
Fe1—C20—C16	70.42 (11)	C216—C215—H215	120.00
Fe1—C20—C19	69.83 (12)	C211—C216—H216	120.00
C16—C20—C19	108.47 (18)	C215—C216—H216	120.00
C112—C111—C116	119.39 (18)	C221—C222—H222	120.00
P1—C111—C112	118.75 (15)	C223—C222—H222	120.00
P1—C111—C116	121.66 (14)	C222—C223—H223	120.00
C111—C112—C113	120.07 (19)	C224—C223—H223	120.00
C112—C113—C114	120.12 (19)	C223—C224—H224	120.00
C113—C114—C115	120.1 (2)	C225—C224—H224	120.00
C114—C115—C116	120.0 (2)	C224—C225—H225	120.00
C111—C116—C115	120.24 (19)	C226—C225—H225	120.00
C122—C121—C126	119.74 (18)	C221—C226—H226	120.00
P1—C121—C122	119.88 (15)	C225—C226—H226	120.00
P1—C121—C126	120.39 (16)	N3—C261—H261	120.00
C121—C122—C123	120.2 (2)	C26—C261—H261	120.00
C122—C123—C124	119.9 (2)	N4—C262—H26A	109.00
C123—C124—C125	120.2 (2)	N4—C262—H26B	109.00
C124—C125—C126	120.4 (2)	N4—C262—H26C	109.00
C121—C126—C125	119.6 (2)	H26A—C262—H26B	109.00
N1—C161—C16	119.44 (18)	H26A—C262—H26C	109.00
N4—N3—C261	119.70 (17)	H26B—C262—H26C	109.00
C262—N4—C263	116.85 (17)	N4—C263—H26D	109.00
N3—N4—C263	119.14 (17)	N4—C263—H26E	109.00
N3—N4—C262	112.27 (16)	N4—C263—H26F	109.00
C13—C12—H12	126.00	H26D—C263—H26E	109.00
Fe1—C12—H12	126.00	H26D—C263—H26F	109.00
C11—C12—H12	126.00	H26E—C263—H26F	109.00
			
C12—Fe1—C11—P1	116.93 (17)	C22—Fe2—C21—P2	−125.11 (18)
C12—Fe1—C11—C15	−118.30 (16)	C22—Fe2—C21—C25	118.36 (16)
C13—Fe1—C11—P1	154.25 (15)	C23—Fe2—C21—P2	−162.91 (15)
C13—Fe1—C11—C12	37.32 (12)	C23—Fe2—C21—C22	−37.80 (11)
C13—Fe1—C11—C15	−80.98 (12)	C23—Fe2—C21—C25	80.55 (12)
C14—Fe1—C11—P1	−162.37 (15)	C24—Fe2—C21—P2	153.81 (15)
C14—Fe1—C11—C12	80.71 (12)	C24—Fe2—C21—C22	−81.08 (12)
C14—Fe1—C11—C15	−37.59 (12)	C24—Fe2—C21—C25	37.27 (12)
C15—Fe1—C11—P1	−124.77 (17)	C25—Fe2—C21—P2	116.53 (18)
C15—Fe1—C11—C12	118.30 (16)	C25—Fe2—C21—C22	−118.36 (16)
C17—Fe1—C11—P1	−76.4 (2)	C27—Fe2—C21—P2	−77.1 (2)
C17—Fe1—C11—C12	166.70 (17)	C27—Fe2—C21—C22	48.0 (2)
C17—Fe1—C11—C15	48.4 (2)	C27—Fe2—C21—C25	166.33 (17)
C18—Fe1—C11—P1	−41.86 (16)	C28—Fe2—C21—P2	−42.29 (16)
C18—Fe1—C11—C12	−158.78 (12)	C28—Fe2—C21—C22	82.83 (13)
C18—Fe1—C11—C15	82.92 (13)	C28—Fe2—C21—C25	−158.82 (12)
C19—Fe1—C11—P1	0.85 (14)	C29—Fe2—C21—P2	0.31 (15)
C19—Fe1—C11—C12	−116.08 (12)	C29—Fe2—C21—C22	125.42 (12)
C19—Fe1—C11—C15	125.62 (12)	C29—Fe2—C21—C25	−116.23 (12)
C20—Fe1—C11—P1	41.48 (16)	C30—Fe2—C21—P2	40.89 (17)
C20—Fe1—C11—C12	−75.44 (14)	C30—Fe2—C21—C22	166.00 (11)
C20—Fe1—C11—C15	166.25 (11)	C30—Fe2—C21—C25	−75.65 (14)
C11—Fe1—C12—C13	119.44 (17)	C21—Fe2—C22—C23	−118.91 (16)
C13—Fe1—C12—C11	−119.44 (17)	C23—Fe2—C22—C21	118.91 (16)
C14—Fe1—C12—C11	−82.30 (12)	C24—Fe2—C22—C21	82.00 (12)
C14—Fe1—C12—C13	37.14 (12)	C24—Fe2—C22—C23	−36.91 (12)
C15—Fe1—C12—C11	−38.52 (11)	C25—Fe2—C22—C21	38.43 (11)
C15—Fe1—C12—C13	80.92 (13)	C25—Fe2—C22—C23	−80.48 (12)
C16—Fe1—C12—C11	166.93 (11)	C26—Fe2—C22—C21	170.18 (15)
C16—Fe1—C12—C13	−73.63 (15)	C26—Fe2—C22—C23	51.3 (2)
C18—Fe1—C12—C11	46.9 (2)	C27—Fe2—C22—C21	−157.41 (11)
C18—Fe1—C12—C13	166.30 (19)	C27—Fe2—C22—C23	83.68 (13)
C19—Fe1—C12—C11	82.40 (13)	C28—Fe2—C22—C21	−114.64 (12)
C19—Fe1—C12—C13	−158.16 (12)	C28—Fe2—C22—C23	126.46 (12)
C20—Fe1—C12—C11	125.35 (11)	C29—Fe2—C22—C21	−74.76 (14)
C20—Fe1—C12—C13	−115.21 (12)	C29—Fe2—C22—C23	166.33 (12)
C11—Fe1—C13—C12	−37.99 (12)	C21—Fe2—C23—C22	38.26 (11)
C11—Fe1—C13—C14	81.94 (12)	C21—Fe2—C23—C24	−81.85 (12)
C12—Fe1—C13—C14	119.93 (17)	C22—Fe2—C23—C24	−120.10 (16)
C14—Fe1—C13—C12	−119.93 (17)	C24—Fe2—C23—C22	120.10 (16)
C15—Fe1—C13—C12	−82.54 (12)	C25—Fe2—C23—C22	82.74 (12)
C15—Fe1—C13—C14	37.40 (11)	C25—Fe2—C23—C24	−37.36 (11)
C16—Fe1—C13—C12	126.31 (12)	C26—Fe2—C23—C22	−155.67 (11)
C16—Fe1—C13—C14	−113.76 (12)	C26—Fe2—C23—C24	84.23 (13)
C17—Fe1—C13—C12	167.85 (12)	C27—Fe2—C23—C22	−113.28 (12)
C17—Fe1—C13—C14	−72.22 (14)	C27—Fe2—C23—C24	126.62 (12)
C19—Fe1—C13—C12	48.7 (2)	C28—Fe2—C29—C30	−119.31 (17)
C19—Fe1—C13—C14	168.67 (17)	C30—Fe2—C29—C28	119.31 (17)
C20—Fe1—C13—C12	83.44 (13)	C21—Fe2—C30—C26	168.02 (11)
C20—Fe1—C13—C14	−156.63 (11)	C21—Fe2—C30—C29	−72.56 (14)
C11—Fe1—C14—C13	−81.69 (12)	C23—Fe2—C30—C26	46.4 (2)
C11—Fe1—C14—C15	38.21 (11)	C23—Fe2—C30—C29	165.82 (17)
C12—Fe1—C14—C13	−37.26 (11)	C24—Fe2—C30—C26	82.41 (13)
C12—Fe1—C14—C15	82.64 (12)	C24—Fe2—C30—C29	−158.16 (12)
C13—Fe1—C14—C15	119.90 (17)	C25—Fe2—C30—C26	125.50 (12)
C15—Fe1—C14—C13	−119.90 (17)	C25—Fe2—C30—C29	−115.08 (12)
C16—Fe1—C14—C13	84.13 (13)	C26—Fe2—C30—C29	119.43 (17)
C16—Fe1—C14—C15	−155.97 (11)	C27—Fe2—C30—C26	−37.75 (11)
C17—Fe1—C14—C13	126.62 (12)	C27—Fe2—C30—C29	81.68 (13)
C17—Fe1—C14—C15	−113.48 (12)	C28—Fe2—C30—C26	−81.93 (13)
C18—Fe1—C14—C13	167.38 (12)	C28—Fe2—C30—C29	37.50 (12)
C18—Fe1—C14—C15	−72.72 (14)	C29—Fe2—C30—C26	−119.43 (17)
C20—Fe1—C14—C13	51.1 (2)	C24—Fe2—C29—C28	168.25 (17)
C20—Fe1—C14—C15	171.00 (17)	C24—Fe2—C29—C30	48.9 (2)
C11—Fe1—C15—C14	−119.11 (17)	C30—Fe2—C28—C29	−37.60 (12)
C12—Fe1—C15—C11	38.42 (11)	C21—Fe2—C29—C28	−113.48 (13)
C12—Fe1—C15—C14	−80.69 (13)	C21—Fe2—C29—C30	127.21 (12)
C13—Fe1—C15—C11	81.99 (12)	C22—Fe2—C29—C28	−71.50 (15)
C13—Fe1—C15—C14	−37.12 (12)	C22—Fe2—C29—C30	169.20 (11)
C14—Fe1—C15—C11	119.11 (17)	C27—Fe2—C29—C30	−81.50 (13)
C16—Fe1—C15—C11	170.02 (15)	C26—Fe2—C29—C28	81.69 (13)
C16—Fe1—C15—C14	50.9 (2)	C25—Fe2—C29—C28	−156.87 (12)
C17—Fe1—C15—C11	−157.35 (11)	C25—Fe2—C29—C30	83.82 (13)
C17—Fe1—C15—C14	83.54 (14)	C26—Fe2—C29—C30	−37.61 (12)
C18—Fe1—C15—C11	−114.66 (12)	C27—Fe2—C29—C28	37.81 (12)
C18—Fe1—C15—C14	126.24 (13)	C11—P1—C121—C122	67.52 (17)
C19—Fe1—C15—C11	−74.46 (14)	S1—P1—C121—C126	13.91 (17)
C19—Fe1—C15—C14	166.43 (12)	S1—P1—C11—Fe1	−64.28 (12)
C12—Fe1—C16—C17	165.86 (12)	S1—P1—C11—C12	23.51 (18)
C12—Fe1—C16—C20	−74.79 (14)	S1—P1—C11—C15	−156.51 (16)
C12—Fe1—C16—C161	43.5 (2)	C111—P1—C11—Fe1	173.73 (11)
C13—Fe1—C16—C17	124.90 (13)	C111—P1—C11—C12	−98.47 (17)
C13—Fe1—C16—C20	−115.75 (12)	C111—P1—C11—C15	81.51 (19)
C13—Fe1—C16—C161	2.6 (2)	C121—P1—C11—Fe1	62.18 (14)
C14—Fe1—C16—C17	82.24 (14)	C121—P1—C11—C12	149.97 (16)
C14—Fe1—C16—C20	−158.41 (12)	C121—P1—C11—C15	−30.1 (2)
C14—Fe1—C16—C161	−40.1 (2)	S1—P1—C111—C112	66.79 (17)
C15—Fe1—C16—C17	46.9 (2)	S1—P1—C111—C116	−107.99 (17)
C15—Fe1—C16—C20	166.25 (16)	C11—P1—C111—C112	−170.51 (16)
C15—Fe1—C16—C161	−75.4 (3)	C11—P1—C111—C116	14.71 (19)
C17—Fe1—C16—C20	119.35 (17)	C121—P1—C111—C112	−57.20 (18)
C17—Fe1—C16—C161	−122.3 (2)	C121—P1—C111—C116	128.03 (17)
C18—Fe1—C16—C17	−38.02 (13)	S1—P1—C121—C122	−166.75 (13)
C18—Fe1—C16—C20	81.33 (13)	C11—P1—C121—C126	−111.82 (16)
C18—Fe1—C16—C161	−160.4 (2)	C111—P1—C121—C122	−44.27 (17)
C19—Fe1—C16—C17	−81.78 (13)	C111—P1—C121—C126	136.39 (15)
C19—Fe1—C16—C20	37.57 (12)	S2—P2—C21—Fe2	−61.59 (13)
C19—Fe1—C16—C161	155.9 (2)	S2—P2—C21—C22	−156.14 (16)
C20—Fe1—C16—C17	−119.35 (17)	S2—P2—C21—C25	27.60 (18)
C20—Fe1—C16—C161	118.3 (2)	C221—P2—C21—Fe2	64.75 (14)
C11—Fe1—C17—C16	168.28 (16)	C221—P2—C21—C22	−29.8 (2)
C11—Fe1—C17—C18	49.5 (2)	C221—P2—C21—C25	153.94 (16)
C13—Fe1—C17—C16	−75.05 (15)	S2—P2—C211—C212	68.84 (18)
C13—Fe1—C17—C18	166.18 (13)	S2—P2—C211—C216	−107.79 (18)
C14—Fe1—C17—C16	−115.15 (12)	C21—P2—C211—C212	−168.37 (17)
C14—Fe1—C17—C18	126.08 (14)	C21—P2—C211—C216	15.0 (2)
C15—Fe1—C17—C16	−157.72 (11)	C221—P2—C211—C212	−55.18 (19)
C15—Fe1—C17—C18	83.50 (15)	C221—P2—C211—C216	128.19 (19)
C16—Fe1—C17—C18	−118.78 (18)	S2—P2—C221—C222	14.71 (17)
C18—Fe1—C17—C16	118.78 (18)	S2—P2—C221—C226	−165.59 (13)
C19—Fe1—C17—C16	81.30 (13)	C21—P2—C221—C222	−111.24 (16)
C19—Fe1—C17—C18	−37.48 (13)	C21—P2—C221—C226	68.46 (17)
C20—Fe1—C17—C16	37.62 (12)	C211—P2—C221—C222	138.02 (15)
C20—Fe1—C17—C18	−81.16 (14)	C211—P2—C221—C226	−42.27 (17)
C11—Fe1—C18—C17	−156.76 (13)	C211—P2—C21—C25	−94.89 (17)
C11—Fe1—C18—C19	83.82 (14)	C211—P2—C21—C22	81.37 (19)
C12—Fe1—C18—C17	169.97 (18)	C211—P2—C21—Fe2	175.92 (12)
C12—Fe1—C18—C19	50.6 (2)	C161—N1—N2—C163	−159.13 (19)
C14—Fe1—C18—C17	−73.02 (15)	C161—N1—N2—C162	−25.9 (3)
C14—Fe1—C18—C19	167.56 (11)	N2—N1—C161—C16	178.84 (17)
C15—Fe1—C18—C17	−113.55 (13)	P1—C11—C12—Fe1	−119.99 (14)
C15—Fe1—C18—C19	127.03 (12)	Fe1—C11—C12—C13	−59.63 (14)
C16—Fe1—C18—C17	37.95 (13)	C15—C11—C12—C13	0.4 (2)
C16—Fe1—C18—C19	−81.47 (13)	Fe1—C11—C15—C14	59.67 (14)
C17—Fe1—C18—C19	−119.42 (18)	P1—C11—C15—Fe1	119.92 (16)
C19—Fe1—C18—C17	119.42 (18)	P1—C11—C15—C14	179.59 (15)
C20—Fe1—C18—C17	81.86 (14)	C12—C11—C15—Fe1	−60.10 (13)
C20—Fe1—C18—C19	−37.56 (12)	P1—C11—C12—C13	−179.62 (14)
C11—Fe1—C19—C18	−113.82 (12)	C15—C11—C12—Fe1	60.03 (13)
C11—Fe1—C19—C20	126.83 (12)	C12—C11—C15—C14	−0.4 (2)
C12—Fe1—C19—C18	−157.11 (12)	C11—C12—C13—Fe1	58.69 (13)
C12—Fe1—C19—C20	83.55 (13)	C11—C12—C13—C14	−0.2 (2)
C13—Fe1—C19—C18	168.48 (17)	Fe1—C12—C13—C14	−58.91 (15)
C13—Fe1—C19—C20	49.1 (2)	C12—C13—C14—Fe1	58.58 (14)
C15—Fe1—C19—C18	−71.92 (14)	Fe1—C13—C14—C15	−58.63 (14)
C15—Fe1—C19—C20	168.74 (11)	C12—C13—C14—C15	−0.1 (2)
C16—Fe1—C19—C18	81.64 (13)	Fe1—C14—C15—C11	−58.82 (13)
C16—Fe1—C19—C20	−37.71 (12)	C13—C14—C15—Fe1	59.12 (15)
C17—Fe1—C19—C18	37.70 (12)	C13—C14—C15—C11	0.3 (2)
C17—Fe1—C19—C20	−81.64 (13)	C20—C16—C17—C18	1.3 (2)
C18—Fe1—C19—C20	−119.35 (17)	C161—C16—C17—C18	−173.52 (19)
C20—Fe1—C19—C18	119.35 (17)	Fe1—C16—C20—C19	−59.68 (14)
C11—Fe1—C20—C16	167.95 (11)	C161—C16—C17—Fe1	126.8 (2)
C11—Fe1—C20—C19	−72.78 (14)	Fe1—C16—C17—C18	59.71 (14)
C12—Fe1—C20—C16	125.62 (12)	C20—C16—C17—Fe1	−58.44 (14)
C12—Fe1—C20—C19	−115.10 (12)	C161—C16—C20—Fe1	−126.55 (19)
C13—Fe1—C20—C16	82.72 (13)	C161—C16—C20—C19	173.76 (19)
C13—Fe1—C20—C19	−158.00 (12)	Fe1—C16—C161—N1	104.9 (2)
C14—Fe1—C20—C16	47.0 (2)	C17—C16—C161—N1	10.5 (3)
C14—Fe1—C20—C19	166.27 (17)	C20—C16—C161—N1	−163.44 (19)
C16—Fe1—C20—C19	119.27 (17)	C17—C16—C20—Fe1	58.46 (14)
C17—Fe1—C20—C16	−37.66 (11)	C17—C16—C20—C19	−1.2 (2)
C17—Fe1—C20—C19	81.61 (13)	Fe1—C17—C18—C19	59.32 (15)
C18—Fe1—C20—C16	−81.69 (12)	C16—C17—C18—C19	−0.9 (2)
C18—Fe1—C20—C19	37.59 (12)	C16—C17—C18—Fe1	−60.17 (14)
C19—Fe1—C20—C16	−119.27 (17)	C17—C18—C19—Fe1	−59.23 (15)
C28—Fe2—C23—C22	−72.53 (14)	Fe1—C18—C19—C20	59.32 (15)
C28—Fe2—C23—C24	167.37 (12)	C17—C18—C19—C20	0.1 (2)
C30—Fe2—C23—C22	171.77 (16)	C18—C19—C20—Fe1	−59.35 (15)
C30—Fe2—C23—C24	51.7 (2)	Fe1—C19—C20—C16	60.05 (14)
C21—Fe2—C24—C23	81.85 (12)	C18—C19—C20—C16	0.7 (2)
C21—Fe2—C24—C25	−37.96 (11)	P1—C111—C116—C115	173.42 (18)
C22—Fe2—C24—C23	37.39 (11)	C116—C111—C112—C113	0.9 (3)
C22—Fe2—C24—C25	−82.42 (12)	C112—C111—C116—C115	−1.3 (3)
C23—Fe2—C24—C25	−119.81 (16)	P1—C111—C112—C113	−174.01 (17)
C25—Fe2—C24—C23	119.81 (16)	C111—C112—C113—C114	0.6 (3)
C26—Fe2—C24—C23	−113.50 (12)	C112—C113—C114—C115	−1.7 (4)
C26—Fe2—C24—C25	126.69 (12)	C113—C114—C115—C116	1.2 (4)
C27—Fe2—C24—C23	−72.05 (14)	C114—C115—C116—C111	0.3 (4)
C27—Fe2—C24—C25	168.14 (12)	P1—C121—C122—C123	−177.75 (15)
C29—Fe2—C24—C23	169.14 (17)	C126—C121—C122—C123	1.6 (3)
C29—Fe2—C24—C25	49.3 (2)	P1—C121—C126—C125	177.24 (15)
C30—Fe2—C24—C23	−156.27 (11)	C122—C121—C126—C125	−2.1 (3)
C30—Fe2—C24—C25	83.92 (13)	C121—C122—C123—C124	−0.2 (3)
C21—Fe2—C25—C24	119.53 (17)	C122—C123—C124—C125	−0.6 (3)
C22—Fe2—C25—C21	−38.45 (11)	C123—C124—C125—C126	0.1 (3)
C22—Fe2—C25—C24	81.09 (12)	C124—C125—C126—C121	1.3 (3)
C23—Fe2—C25—C21	−82.39 (12)	C261—N3—N4—C262	−162.1 (2)
C23—Fe2—C25—C24	37.14 (12)	C261—N3—N4—C263	−20.2 (3)
C24—Fe2—C25—C21	−119.53 (17)	N4—N3—C261—C26	−178.36 (18)
C26—Fe2—C25—C21	167.24 (11)	Fe2—C21—C22—C23	59.63 (13)
C26—Fe2—C25—C24	−73.23 (14)	P2—C21—C22—Fe2	123.05 (16)
C28—Fe2—C25—C21	46.7 (2)	P2—C21—C22—C23	−177.33 (15)
C28—Fe2—C25—C24	166.26 (19)	C25—C21—C22—Fe2	−60.26 (13)
C29—Fe2—C25—C21	82.46 (13)	C25—C21—C22—C23	−0.6 (2)
C29—Fe2—C25—C24	−158.01 (12)	Fe2—C21—C25—C24	−59.50 (13)
C30—Fe2—C25—C21	125.49 (11)	P2—C21—C25—Fe2	−122.81 (14)
C30—Fe2—C25—C24	−114.98 (12)	P2—C21—C25—C24	177.70 (14)
C22—Fe2—C26—C27	46.7 (2)	C22—C21—C25—Fe2	60.22 (13)
C22—Fe2—C26—C30	165.86 (16)	C22—C21—C25—C24	0.7 (2)
C22—Fe2—C26—C261	−74.9 (3)	Fe2—C22—C23—C24	59.05 (14)
C23—Fe2—C26—C27	82.29 (14)	C21—C22—C23—Fe2	−58.75 (13)
C23—Fe2—C26—C30	−158.57 (12)	C21—C22—C23—C24	0.3 (2)
C23—Fe2—C26—C261	−39.3 (2)	Fe2—C23—C24—C25	58.68 (14)
C24—Fe2—C26—C27	124.84 (13)	C22—C23—C24—Fe2	−58.53 (14)
C24—Fe2—C26—C30	−116.02 (12)	C22—C23—C24—C25	0.2 (2)
C24—Fe2—C26—C261	3.3 (2)	Fe2—C24—C25—C21	58.48 (13)
C25—Fe2—C26—C27	165.68 (12)	C23—C24—C25—Fe2	−59.02 (14)
C25—Fe2—C26—C30	−75.18 (14)	C23—C24—C25—C21	−0.5 (2)
C25—Fe2—C26—C261	44.1 (2)	Fe2—C26—C27—C28	59.79 (14)
C27—Fe2—C26—C30	119.14 (17)	C30—C26—C27—Fe2	−58.27 (14)
C27—Fe2—C26—C261	−121.6 (2)	C30—C26—C27—C28	1.5 (2)
C28—Fe2—C26—C27	−38.12 (13)	C261—C26—C27—Fe2	124.1 (2)
C28—Fe2—C26—C30	81.02 (13)	C261—C26—C27—C28	−176.1 (2)
C28—Fe2—C26—C261	−159.7 (2)	Fe2—C26—C30—C29	−59.54 (14)
C29—Fe2—C26—C27	−81.68 (13)	C27—C26—C30—Fe2	58.11 (14)
C29—Fe2—C26—C30	37.46 (12)	C27—C26—C30—C29	−1.4 (2)
C29—Fe2—C26—C261	156.7 (2)	C261—C26—C30—Fe2	−124.2 (2)
C30—Fe2—C26—C27	−119.14 (17)	C261—C26—C30—C29	176.23 (19)
C30—Fe2—C26—C261	119.3 (2)	Fe2—C26—C261—N3	103.6 (2)
C21—Fe2—C27—C26	168.74 (16)	C27—C26—C261—N3	11.6 (3)
C21—Fe2—C27—C28	50.0 (2)	C30—C26—C261—N3	−165.6 (2)
C22—Fe2—C27—C26	−157.63 (11)	Fe2—C27—C28—C29	59.57 (15)
C22—Fe2—C27—C28	83.64 (15)	C26—C27—C28—Fe2	−60.63 (14)
C23—Fe2—C27—C26	−114.93 (12)	C26—C27—C28—C29	−1.1 (2)
C23—Fe2—C27—C28	126.34 (14)	Fe2—C28—C29—C30	59.50 (15)
C24—Fe2—C27—C26	−74.99 (15)	C27—C28—C29—Fe2	−59.32 (15)
C24—Fe2—C27—C28	166.28 (13)	C27—C28—C29—C30	0.2 (2)
C26—Fe2—C27—C28	−118.73 (19)	Fe2—C29—C30—C26	60.13 (14)
C28—Fe2—C27—C26	118.73 (19)	C28—C29—C30—Fe2	−59.35 (15)
C29—Fe2—C27—C26	81.39 (13)	C28—C29—C30—C26	0.8 (2)
C29—Fe2—C27—C28	−37.34 (14)	P2—C211—C212—C213	−176.21 (18)
C30—Fe2—C27—C26	37.73 (12)	C216—C211—C212—C213	0.5 (3)
C30—Fe2—C27—C28	−81.01 (14)	P2—C211—C216—C215	176.1 (2)
C21—Fe2—C28—C27	−156.45 (13)	C212—C211—C216—C215	−0.5 (4)
C21—Fe2—C28—C29	84.14 (14)	C211—C212—C213—C214	0.0 (4)
C22—Fe2—C28—C27	−113.33 (13)	C212—C213—C214—C215	−0.6 (4)
C22—Fe2—C28—C29	127.26 (12)	C213—C214—C215—C216	0.6 (4)
C23—Fe2—C28—C27	−72.75 (16)	C214—C215—C216—C211	0.0 (4)
C23—Fe2—C28—C29	167.84 (12)	P2—C221—C222—C223	178.39 (15)
C25—Fe2—C28—C27	170.38 (18)	C226—C221—C222—C223	−1.3 (3)
C25—Fe2—C28—C29	51.0 (2)	P2—C221—C226—C225	−178.13 (15)
C26—Fe2—C28—C27	37.91 (13)	C222—C221—C226—C225	1.6 (3)
C26—Fe2—C28—C29	−81.50 (13)	C221—C222—C223—C224	0.1 (3)
C27—Fe2—C28—C29	−119.41 (19)	C222—C223—C224—C225	0.9 (3)
C29—Fe2—C28—C27	119.41 (19)	C223—C224—C225—C226	−0.7 (3)
C30—Fe2—C28—C27	81.82 (14)	C224—C225—C226—C221	−0.6 (3)

### Hydrogen-bond geometry (Å, º)

Cg1, Cg2 and Cg3 are the centroids of rings C111–C116, C16–C20 and C26–C30, respectively.

**Table:**

*D*—H···*A*	*D*—H	H···*A*	*D*···*A*	*D*—H···*A*
C123—H123···N4	0.95	2.62	3.373 (3)	136
C226—H226···S1^i^	0.95	2.77	3.416 (2)	126
C262—H26*A*···*Cg*1^ii^	0.98	2.91	3.878	172
C125—H125···*Cg*2^i^	0.95	2.80	3.533	135
C223—H223···*Cg*3^iii^	0.95	2.70	3.478	139

Symmetry codes: (i) −*x*, −*y*+1, −*z*+1; (ii) −*x*, −*y*, −*z*+2; (iii) −*x*−1, −*y*, −*z*+1.
